# The Role of Inflammation Resolution Speed in Airway Smooth Muscle Mass Accumulation in Asthma: Insight from a Theoretical Model

**DOI:** 10.1371/journal.pone.0090162

**Published:** 2014-03-14

**Authors:** Igor L. Chernyavsky, Huguette Croisier, Lloyd A. C. Chapman, Laura S. Kimpton, Jonathan E. Hiorns, Bindi S. Brook, Oliver E. Jensen, Charlotte K. Billington, Ian P. Hall, Simon R. Johnson

**Affiliations:** 1 School of Mathematical Sciences, University of Nottingham, Nottingham, United Kingdom; 2 Mathematical Institute, University of Oxford, Oxford, United Kingdom; 3 School of Mathematics, University of Manchester, Manchester, United Kingdom; 4 Department of Therapeutics and Molecular Medicine, University of Nottingham, Nottingham, United Kingdom; SUNY College of Nanoscale Science and Engineering, United States of America

## Abstract

Despite a large amount of *in vitro* data, the dynamics of airway smooth muscle (ASM) mass increase in the airways of patients with asthma is not well understood. Here, we present a novel mathematical model that describes qualitatively the growth dynamics of ASM cells over short and long terms in the normal and inflammatory environments typically observed in asthma. The degree of ASM accumulation can be explained by an increase in the rate at which ASM cells switch between non-proliferative and proliferative states, driven by episodic inflammatory events. Our model explores the idea that remodelling due to ASM hyperplasia increases with the frequency and magnitude of these inflammatory events, relative to certain sensitivity thresholds. It highlights the importance of inflammation resolution speed by showing that when resolution is slow, even a series of small exacerbation events can result in significant remodelling, which persists after the inflammatory episodes. In addition, we demonstrate how the uncertainty in long-term outcome may be quantified and used to design an optimal low-risk individual anti-proliferative treatment strategy. The model shows that the rate of clearance of ASM proliferation and recruitment factors after an acute inflammatory event is a potentially important, and hitherto unrecognised, target for anti-remodelling therapy in asthma. It also suggests new ways of quantifying inflammation severity that could improve prediction of the extent of ASM accumulation. This ASM growth model should prove useful for designing new experiments or as a building block of more detailed multi-cellular tissue-level models.

## Introduction

Asthma is a chronic inflammatory disease, characterised by acute inflammatory events during which antigen exposure triggers the production or recruitment of inflammatory cells (mast cells, T-cells, eosinophils, etc.). These cells secrete inflammatory mediators and growth factors which induce acute inflammation and bronchoconstriction of an airway wall over the short term (i.e. minutes to hours) and its remodelling over the long term (i.e. days to months) [1], [2].

**Figure 1:**
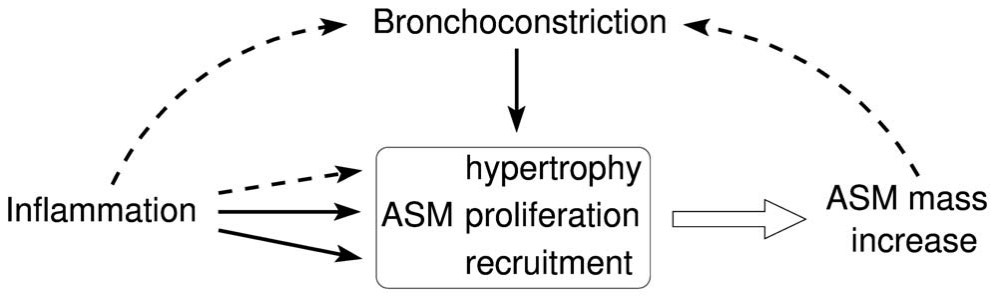
Possible pathways of chronic ASM mass accumulation in asthma (solid lines indicate the mechanisms included in the mathematical model).

Airway smooth muscle (ASM) mass increase is an important aspect of both airway remodelling and hyper-responsiveness [3], [4]. ASM mass accumulation can be triggered by multiple factors, including inflammation and bronchoconstriction (see Fig. 1). There is ongoing debate regarding the relative contributions of ASM cell proliferation, hypertrophy and recruitment (e.g. via differentiated fibroblasts and myofibroblasts) in asthmatic airways [5]–[7]. A large number of inflammatory mediators are associated with ASM proliferation *in vitro*, and infiltrating inflammatory cells are a feature of asthma [8]. However, the precise role of these factors in ASM mass accumulation *in vivo* is not well understood [7]. The many factors involved in airway remodelling, combined with the difficulties and risks of invasive airway biopsies in patients with asthma (particularly during exacerbations) and the lack of consensus on animal models and *in vitro* systems, have motivated research on understanding ASM mass increase by non-invasive means, including mathematical modelling [9], [10].

Quantitative data on various aspects of lung function and airway inflammation can be used to inform mathematical models. These include [11]: (i) bronchial biopsies, which reveal the amount of airway wall remodelling and allow one to estimate the percentage of proliferating and apoptotic ASM cells; (ii) analyses of inflammatory mediators present in blood and airway sputum differential cell counts; (iii) exhaled nitric oxide concentration measurements, and (iv) lung function tests, in particular forced expiratory volume in one second (FEV_1_) recordings. The most direct quantitative information on ASM accumulation, provided by bronchial biopsies, is very limited due to the invasive nature of this procedure. Lung function and inflammatory biomarker measures, however, are more readily performed.

In this paper, we focus on developing a simple theoretical model of long-term ASM hyperplasia (proliferation and recruitment) via inflammatory pathways, which has the potential to integrate the information from airway biopsies and inflammatory biomarkers. This model allows possible ASM accumulation scenarios to be explored and suggests possible new targets for diagnosis and prevention of ASM remodelling.

## Methods

### Model formulation and assumptions

**Figure 2:**
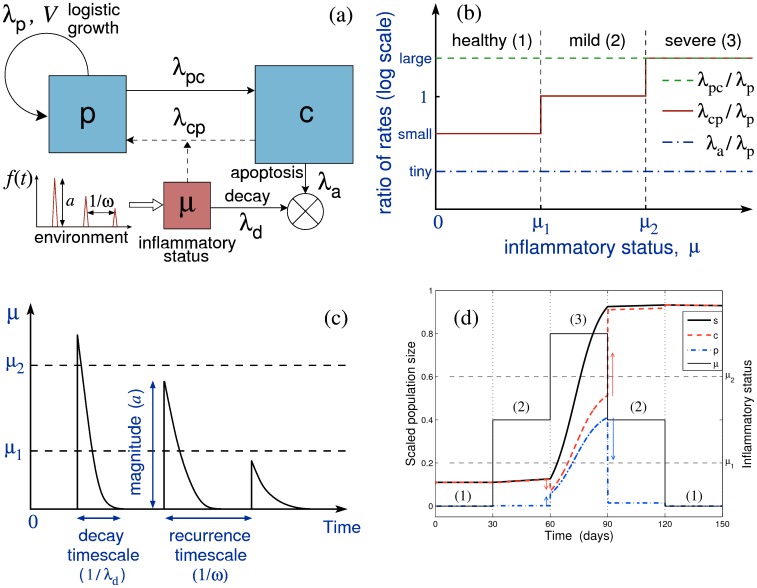
A schematic of the model design. (a) Schematic representation of the model with *p* being the amount of ASM cells in proliferating state, *c* the amount of non-proliferative cells and μ the inflammatory status; λ_p_ is the proliferation rate, λ_a_ is the apoptosis rate, λ_cp_ and λ_pc_ are the switching rates between non-proliferative and proliferative states, λ_d_ is the inflammation clearance rate, and *f*(*t*) is a time-dependent external inflammatory stimulus. (b) Dependence of the model parameters on the inflammatory status μ (three levels of inflammation are characterised by the thresholds μ_1_ and μ_2_; see Table 1 for reference values). Rates are plotted on a logarithmic vertical scale. (c) An illustration of the inflammatory status dynamics induced by a series of environmental stimuli such as shown in (a), illustrating graphically the parameters λ_d_, *a*, and ω. (d) A simulation of the ASM cell population response (*p*, blue dash-dotted; *c*, red dashed; *s* = *p*+*c*, thick black solid) to a stepwise variation in the inflammation status (thin solid); the arrows show the direction of change in the ASM subpopulations. Although the inflammatory status returns to its initial state at the end of the simulation, the total ASM cell population has irreversibly increased, showing thereby “effective” hysteresis. Only the time spent in “severe” regime (μ>μ_2_) contributes to substantial growth (over weeks); however, the “moderate” regime (μ_1_<μ<μ_2_) can also give rise to substantial growth over a longer timescale (months). Note that the proportion of proliferative cells (blue dash-dotted) is significant only during the “severe inflammation” regime (3).

We employ a simple model, schematised in Fig. 2(a), in which we assume ASM cells are either in a proliferative state (which also accounts for recruitment) or a non-proliferative state [12], [13]. These are respectively characterized by time-dependent population sizes in an airway wall *p*(*t*) and *c*(*t*), and the total ASM cell population is denoted *s*(*t*) = *p*+*c*. In the following, “proliferation” refers to both cell replication and recruitment, i.e. it is used as a synonym for hyperplasia.

Switching between the two states is governed by the rates λ_cp_ and λ_pc_. Proliferation of the *p*-subpopulation is assumed to follow a logistic law (see S1.1 in Materials S1), with proliferation rate λ_p_, and maximal population size (“total capacity”) *V.* The latter implicitly includes the effect of spatial constraints in the airway wall, as well as *p*-cell apoptosis. For convenience, in the following, all population sizes are normalised by *V*, so that the maximum total cell population size is *s* = 1. The apoptosis rate of the *c*-subpopulation, λ_a_, is assumed to be much smaller than λ_p_ (λ_a_<<λ_p_) [14]. We also assume that ASM cells do not remain in the proliferative state for a long time (λ_pc_>>λ_p_).

A third variable, the *inflammatory status* μ(*t*), modulates the switching rate λ_cp_ of ASM cells between the non-proliferative and proliferative states (Fig. 2(a,b)). We assume that μ changes in response to environmental stimuli of average magnitude *a* and average frequency ω and decays with a rate λ_d_ (the inflammation clearance rate), as illustrated in Fig. 2(c) (see Materials S1 for governing equations). The terminology “inflammatory status” refers in this work to all possible downstream triggers of ASM mass increase, including bronchoconstriction [15], [16] (see Fig. 1). Similarly, “exacerbation”, or “acute inflammatory event”, refers to any substantial rapid increase in the inflammatory status (whether or not this leads to patient hospitalisation).

The way the switching rate λ_cp_ varies as a function of μ, relative to the other rate parameters, is depicted in Fig. 2(b). It is characterised by two thresholds, μ_1_ and μ_2_, such that:

1. For μ<μ_1_ (the “healthy” case), λ_cp_ is sufficiently small (λ_a_<<λ_cp_<<λ_p_<<λ_pc_) for the total ASM cell population to remain approximately stationary (case (1) in Fig. 2(b,d));
2. For μ_1_≤μ<μ_2_ (“mild” inflammation), λ_cp_ is elevated (λ_a_<<λ_cp_≈λ_p_<<λ_pc_) such that the ASM cell population increases on a slow ‘remodelling’ timescale (*t*≈*T*>>1/λ_p_, i.e. weeks to months; case (2) in Fig. 2(b,d));
3. For μ*≥*μ_2_ (“severe” inflammation), λ_cp_ is increased further (λ_a_<<λ_p_<<λ_cp_≈λ_pc_), allowing ASM cell population to increase on a fast ‘proliferation’ timescale (*t*≈1/λ_p_, i.e. days to weeks; case (3) in Fig. 2(b,d)).

Table 1 gives the literature-based values of the model parameters.

**Table 1:** Literature-based and estimated parameter values used in the ASM growth model.

Parameter	Value	Reference
ASM cell proliferation rate (λ_p_)^ *^	∼1/3 days^−1^	[5], [7]
Remodelling time scale (*T*)	∼100 days	[2]
Time-scale parameter (ε = 1/(λ_p_ *T*))	0.02	
Relative *p*→*c* switching rate (λ_pc_/λ_p_)	50 (∼ε^−1^)	
Relative *c*→*p* switching rate (λ_cp_/λ_p_)	0.016 (∼ε) for μ<μ_1,_	
	0.8 (∼1) for μ_1_<μ<μ_2,_	
	40 (∼ε^−1^) for μ>μ_2_	
Relative ASM apoptosis rate (λ_a_/λ_p_)	4×10^−4^ (∼ε^2^)	
Relative severe inflammatory threshold (μ_2_/μ_1_)	2.5	
Relative inflammatory stimulus magnitude (*a*/μ_1_)	0.1–10	
Relative frequency of inflammatory events (ω/λ_p_)	0.1–1 (monthly – weekly)	
Inflammation resolution rate IR = λ_d_/λ_p_	0.1–10	

(^*^ indicates the values estimated from *in vitro* experiments.)

We consider both periodic and irregular timing of exacerbation events in the simulations. Given the irregular nature of events that are likely to occur in reality, we seek to explore the impact of this randomness on ASM remodelling. We run a large number of simulations (*N* = 1000), in each of which we allow exacerbation events to occur randomly according to a Poisson process. The resulting fold-increase in ASM mass at 300 days is recorded for each simulation. This may be interpreted as 1000 possible outcomes in an individual subject given the same average characteristics (magnitude and mean frequency) from a given initial state. The outcomes plotted as a histogram then allow quantification of the likelihood of developing severely or moderately remodelled airways. This is analogous to a weather forecast in which a number of different possible outcomes are determined from a given current state.

### Solution techniques

We use a combination of analytical techniques (multi-timescale analysis) and computer simulations (with *ode45* MATLAB Runge-Kutta solver) to characterise the dynamics of the ASM cell population growth (see S1.2 in Materials S1 for technical details).

## Results

### Survey of ASM growth scenarios

The fundamental properties of the model are illustrated in Figure 2(d), where the effect of successive stepwise increases and decreases of the inflammation status μ through the three inflammation regimes (Fig. 2b) is simulated. Over the considered time span (about 1 month in each regime), only severe inflammation (case (3)) leads to substantial growth of the ASM cell population. In the other two cases, the non-proliferative phenotype is predominant and population growth is either negligible (case (1)) or very slow (case (2)). As a consequence, despite μ returning to the initial non-inflammatory value at the end of the considered period (5 months), the ASM cell population size has not decreased to its original value. This feature results from the existence of different timescales in the model and accounts for the persistence of ASM remodelling in asthma beyond acute phases of inflammation (in agreement with a recent chronically-challenged mouse model of asthma [17]). Note that the theoretical model allows for the existence of a very slow decrease in ASM mass in the case of prolonged absence of hyperplastic stimulus (over a timescale of years, thus not apparent in Fig. 2d). Indeed, our model suggests that there is a dynamic balance between state-switching, proliferation and apoptosis in a population of ASM cells: λ_cp_/λ_pc_ ∼ λ_a_/λ_p_ (see S1.2 in Materials S1). Such a slow decay in ASM mass has been observed recently in an equine model of asthma (see Discussion and [18]).

### Recurrent inflammatory episodes

**Figure 3:**
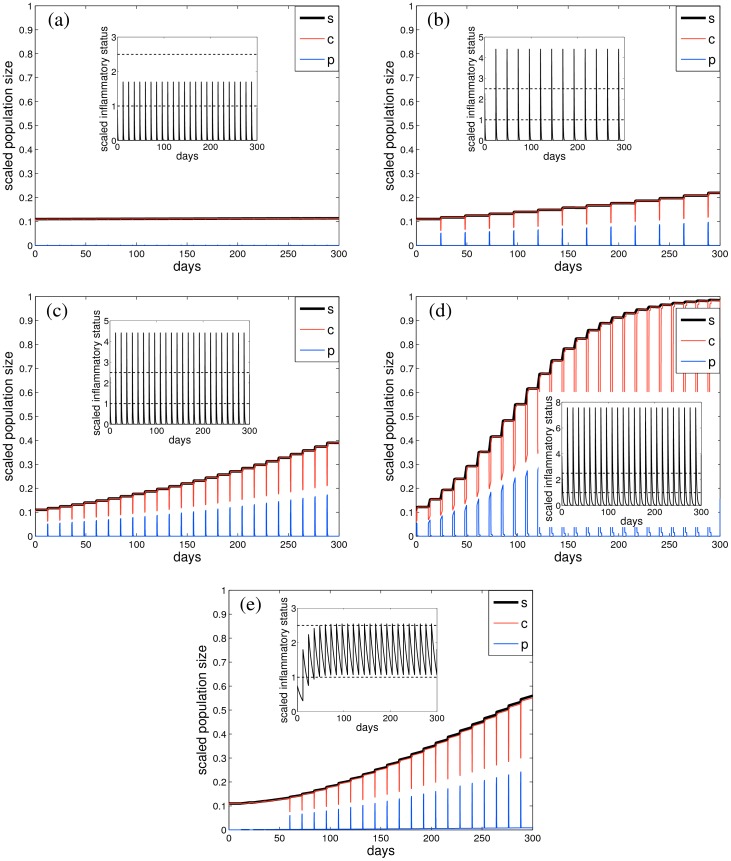
Representative ASM cell population growth dynamics over a period of 300 days, starting from a “healthy” state (*c* = 0.1, *p* = 0.01), for different values of normalised inflammation magnitude *a*/μ_1_, frequency ω/λ_p_ and inflammation resolution rate λ_d_/λ_p_: *a*/μ_1_  =  {2 (a), 5 (b,c), 8 (d), 1.5 (e)}, ω/λ_p_  =  {0.25 (a,c–e), 0.125 (b)} and λ_d_/λ_p_  =  {7 (a), 5 (b,c), 2 (d), 0.22 (e)}. (a,b) show no substantial ASM accumulation, (c,e) show “moderate” and (d) “severe” ASM hyperplasia. The amount of *c*-cells is depicted by the red line, *p*-cells (blue) and total population size *s* = *p*+*c* (thick black). Insets plot the corresponding inflammatory status μ (solid black) and the inflammation level thresholds (horizontal dashed lines).

Figure 3 shows examples of the different proliferation scenarios that can be encountered under a series of regular inflammatory events distributed over about one year, depending on the inflammation magnitude, frequency and resolution rate. When the magnitude of inflammatory events is below the “severe” inflammation threshold μ_2_ and the rate of resolution is high, there is no significant ASM mass increase (Fig. 3a); more severe inflammatory events, depending on their magnitude, lead to “moderate” (Fig. 3c) or “severe” (Fig. 3d) long-term ASM mass accumulation. Similarly, changing the frequency of the events from monthly (Fig. 3b) to semi-monthly (Fig. 3c), for the same magnitude and resolution rate, results in about two-fold ASM mass increase.

The outcome of the exacerbation events depends strongly on whether successive inflammatory events accumulate to increase the mean inflammatory status (Fig. 3e) or are independent (Fig. 3(a–d)). In particular, although the frequency and magnitude of inflammatory events are nearly identical in Figs 3(a) and 3(e), the ASM mass remains unchanged in Fig. 3(a) while it increases substantially in Fig. 3(e), due to the slow inflammation resolution.

**Figure 4:**
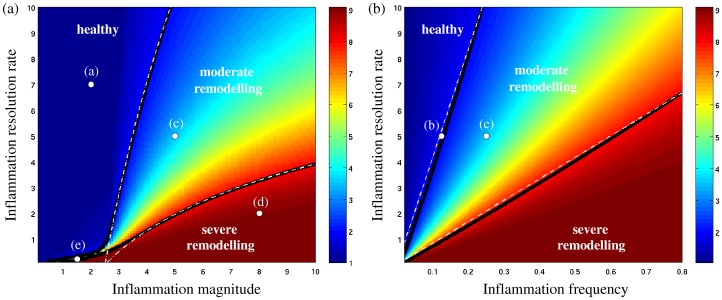
Survey of ASM growth scenarios, showing fold-increase in ASM population size after 300 days (colour scale) as a function of the inflammation resolution rate IR  =  λ_d_/λ_p_ and (a) inflammation magnitude *a*/μ_1_ (for fixed frequency ω/λ_p_  = 0.25) or (b) inflammation frequency ω/λ_p_ (for fixed magnitude *a*/μ_1_  = 5). White dots indicate the growth regimes shown in Fig. 2. Solid black lines are the computed isolines of the 2- and 8-fold ASM growth, which agree with the theoretically predicted dependence λ_d_ ∼ ω log *a*/μ_2_ (dashed white lines; see Materials S1).

Figure 4 summarises the possible growth scenarios illustrated in Fig. 3(a–e). The relative fold-increase in ASM population size reached at the end of the observation period (300 days) is plotted as a function of the normalised inflammation resolution rate IR  = λ_d_/λ_p_ (vertical axis), and (on the horizontal axis) the normalised inflammation magnitude *a/*μ_1_ (Fig. 4a) or frequency ω/λ_p_ (Fig. 4b).

When the inflammation resolution is very slow (small IR <<1), the inflammatory status μ accumulates even if individual exacerbations are mild and infrequent, leading to severe increase in ASM mass for a wide range of magnitudes and frequencies (red zones in Fig. 4). Defining the boundaries between “healthy”, “moderate” and “severe” outcomes in terms of the isolines for a given long-term fold-increase in ASM mass (the two thick solid lines in Fig. 4(a), corresponding respectively to 2- and 8-fold increase), we see that the chance of an individual being in the “moderate” ASM growth regime, as either the exacerbation magnitude or frequency are increased, drops considerably when the inflammation resolution is slow (IR <1), indicating the possibility of a catastrophic shift straight from the “healthy” to the “severe remodelling” scenario when the inflammation resolution is not fast enough.

For very rapid inflammation resolution (high IR >>1), when the individual inflammatory episodes are almost independent of each other, the long-term ASM mass accumulation is determined solely by the total time spent with inflammatory status above the “severe” inflammation threshold (μ*≥*μ_2_). Our analysis (see S1.2 in Materials S1) shows that this time is linearly proportional to the exacerbation frequency ω, but the dependence on the relative inflammatory event magnitude *a/*μ_2_ is nonlinear. Hence, the isolines of constant ASM growth are expected to obey IR ∼ (ω/λ_p_) log(*a/*μ_2_) (white dashed lines in Fig. 4; see also S1.2 in Materials S1). This theoretical prediction agrees well with the computed growth isolines (thick black lines in Fig. 4) for IR >1. The model therefore suggests an integral *inflammation severity* (IS) index
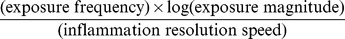
(where exposure magnitude is measured relative to the threshold), which has the potential to be used as a predictor of long-term ASM remodelling in the case of moderate-to-fast inflammation resolution. In other words, given the time series of a biomarker of inflammation for a cohort of patients, the model indicates that the IS index computed from these series could correlate with the degree of ASM accumulation in this group.

### Individual history effects in ASM growth

For slow recovery from inflammation (IR <1), substantial ASM mass growth can occur even if the inflammatory magnitude lies below the “severe” inflammation threshold (*a*<μ_2_; see e.g. Fig. 3e), in contrast to the fast IR case which requires *a*>μ_2_ for substantial growth. Therefore, the individual history of inflammatory, pro-remodelling events can have a very strong impact on the degree of long-term ASM hyperplasia when successive events can accumulate.

**Figure 5:**
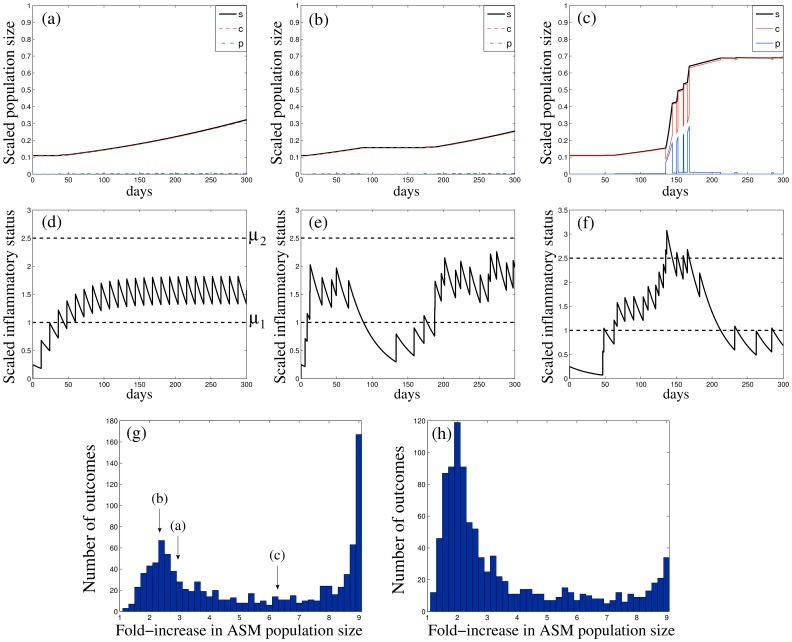
The role of individual inflammation history in the case of slow inflammation resolution (IR <<1). (a–c) ASM population size dynamics (*c*-cells, red; *p*-cells, blue; total population *s*, black) and (d–f) the corresponding inflammatory status evolution (μ, solid black; inflammatory thresholds μ_1_ and μ_2_, dashed), characterized by the same inflammation resolution rate, magnitude and average stimulus frequency (λ_d_/λ_p_  = 0.08, *a*/μ_1_  = 0.5, ω/λ_p_  = 0.25). (d) Regular series of inflammatory events; (e–f) two realisations of a series of inflammatory events at random times for the same mean frequency (about once a fortnight) as in (d). (g) Distribution of fold-increase in ASM mass after 300 days for a random sequence of inflammatory events with the same characteristics as in panels (b, c); arrows indicate the fold-increase corresponding to (a–c). (h) The distribution of outcomes with an increase of 25% in the inflammation resolution rate (λ_d_/λ_p_  = 0.1). (The outcome histograms (g,h) are computed for *N* = 1000 instances).

Figure 5 illustrates the importance of history in determining the outcome of a series of inflammatory episodes, when the inflammation resolution is slow. For given inflammation magnitude, frequency and resolution rate, the amount of ASM growth elicited by an inflammatory event depends crucially on the timing of the previous inflammatory events. For example, for regular episodes at a frequency of about once a fortnight (Fig. 5d) and small inflammation magnitude, the inflammatory status lies well within the “moderate inflammation” zone after about 40 days, leading to a “moderate” (less than 3-fold) long-term increase in ASM mass (Fig. 5a). However, when the events occur irregularly (Fig. 5(e,f)) with the same average characteristics as in Fig. 5(d), the individual long-term outcome is much more diverse (Fig. 5(b,c)), due to the possibility of accumulated inflammatory events or prolonged periods of remission.

In order to demonstrate the impact of dangerously frequent exacerbations, Figure 5(g) shows the distribution (histogram) of *N* = 1000 possible outcomes in a given individual at the end of an observation period of 300 days (for a series of random events exemplified in Fig. 5(e,f)). The distribution is bimodal (i.e. has two peaks), with one mode being close to the nearly “healthy” state reached in the absence of randomness (Fig. 5(a,d)), and the other mode corresponding to severe increase in ASM mass (Fig. 5(c,f)). Figure 5(h) shows that an increase of only 25% in the inflammation resolution rate IR can substantially push the mean of the distribution toward the “healthy” mode. This emphasises the importance of individual inflammatory history for prognosis and management of ASM hyperplasia.

**Figure 6:**
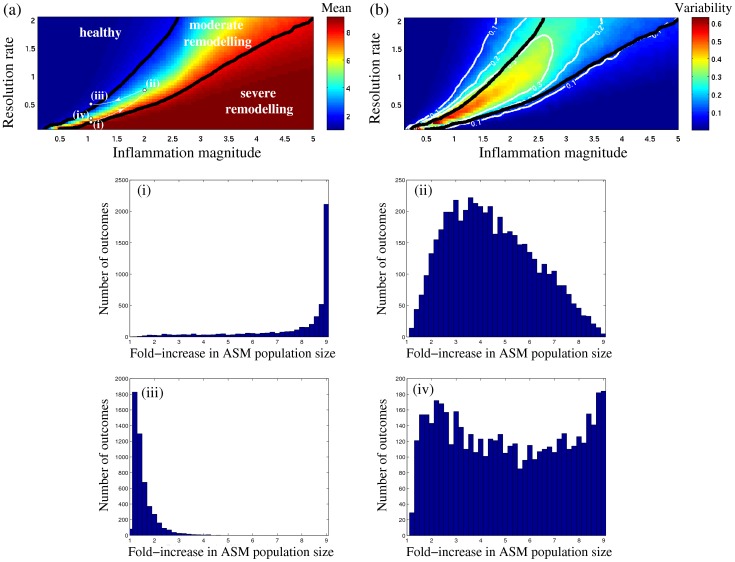
Survey of stochastic ASM growth scenarios in the region of low-to-moderate inflammation resolution rate. (a) Mean fold-increase in the ASM population size at 300 days (colour scale) as a function of inflammation resolution rate IR  = λ_d_/λ_p_ and inflammation magnitude *a*/μ_1_ (*cf*. Fig. 3a). (b) Corresponding uncertainty (colours) quantified as the ratio of SD to the mean; white lines indicate the uncertainty isolines. (Solid black lines in (a,b) show 2- and 8-fold increase in mean; computed for a sample size of *N* = 500 random realisations). The parameter dependence of outcome distributions (*N* = 5000) are illustrated in (i–iv), indicated by white dots in (a). (Note the variation in vertical scales between panels (i, iii) and (ii, iv)). The quickest transition from (i) to (iii) crosses the high uncertainty region ((iv), red in panel (b)), while a trajectory that bypasses it (white line in (a)) protects against “severe” remodelling outcomes.

We further survey the effect of variability in the timing of acute inflammatory stimuli in Figure 6, which is the analogue of Fig. 4(a) for the case of randomly occurring exacerbation events, and is plotted for a whole range of inflammation resolution rates and magnitudes. Each point in Fig. 6(a) represents the mean long-term fold-increase in ASM mass (at the end of 300 days) for given inflammation characteristics, while each point in Fig. 6(b) represents the variability of the outcome (quantified as the ratio of standard deviation to the mean). Some representative distributions of outcomes are shown in Fig. 6(i–iv).

Figure 6(a) indicates that, at slow resolution rates, the “moderate remodelling” transition zone is larger for random inflammatory episodes (Fig. 6a) than for regular episodes (Fig. 4a). Figure 6(b) shows that the variability of “healthy” and “severe remodelling” outcomes (blue zone in Fig. 6(b)) is very low; uncertainty is mainly concentrated in the “moderate remodelling” zone, and rapidly diminishes as resolution speed increases (Fig. 6b).

The high uncertainty region (yellow-to-red, Fig. 6b) is characterised by a very wide distribution of outcomes, even though some of these distributions (e.g. Fig. 6(iv)) have relatively low mean values of long-term ASM growth. These are therefore as undesirable as high-mean distributions (e.g. Fig. 6(i)) due to a high risk of ending up with “severe” remodelling in the long term.

## Discussion

In this work, we have proposed a simple model of inflammation-induced ASM hyperplasia, an important aspect of airway remodelling in asthma. The model assumes that ASM cells can be in one of two states: ‘proliferative’ (also accounting for the recruitment and differentiation of fibroblasts and myofibroblasts by inflammatory mediators [19]) or ‘non-proliferative’. Although the former is usually associated with a synthetic activity, and the latter with a contractile activity, we avoided using this additional terminology here since the synthetic and contractile functions of ASM cells may not be mutually exclusive [20]. Bidirectional switching between the two states is allowed (as observed *in vitro* in both human ASM cells in culture and in human tracheal smooth muscle strips [13], [21]), and it is assumed that the level of the inflammatory status μ controls the rate of switching between the two ASM states via the existence of two sensitivity thresholds (Fig. 2b). With the aid of mathematical techniques (see Materials S1), we have shown that this leads to the existence of three corresponding ASM growth regimes (Fig. 2d) and three qualitatively different remodelling outcomes (Figs 4 and 6), matching the classification commonly used in clinical practice [6], [22].

The existence of different timescales in the model accounts for several important features of asthma:

- the persistence of airway remodelling despite the resolution of acute inflammation [1], [17], and the correlation of ASM mass with asthma severity rather than with asthma duration [23];
- the presence of airway remodelling and abnormal lung function very early in the history of asthma [24];
- the very slow decline (of the order of years) in ASM mass in the continued absence of hyperplastic stimuli [18].

Feature (i) can be directly related to Fig. 2(d); feature (ii) can be explained by μ having crossed the severe inflammation threshold, and remained above it a substantial amount of time, early in the disease history; feature (iii) is consistent with the non-negligible role played by apoptosis when the inflammatory status is kept in the healthy range (Fig. 2b) for a sufficiently long time (longer than that considered in Fig. 2(d); see also S1.2 in Materials S1).

### Cumulative effects of individual inflammatory history at slow resolution rate

Assuming that inflammatory events are characterised by transient increases in the inflammatory status μ (Fig. 2c), our model predicts that long-term ASM growth is essentially controlled by three parameters: the inflammation resolution speed (IR), the inflammation magnitude (relative to the two sensitivity thresholds μ_1_ and μ_2_) and the frequency of inflammatory episodes. The severity and frequency of the inflammatory episodes could be targeted to ensure that an asthmatic individual does not fall into the dangerous “severe remodelling” zone (red in Fig. 4). However, our model indicates that faster inflammation resolution could offer a better protection against severe ASM remodelling by increasing the probability of “healthy state” or “moderate” growth (see Figs 4a and 6a).

In addition, irregularity of the occurrence of inflammatory episodes, together with the existence of sensitivity thresholds, lead to a large variability of growth scenarios (see “moderate remodelling” region in Figs 5(b,c) and 6). Thus, one should take into account not only the expected outcome but also the degree of uncertainty when deciding on the optimal low-risk treatment strategy (see arrows in Fig. 6a). That means it would be desirable to avoid high outcome variability (Fig. 6b) when navigating towards the “healthy” state by varying the inflammation resolution speed, magnitude or frequency. (Note that another possibility would be to modify the landscape of outcomes itself by elevating the inflammation sensitivity threshold μ_2_, thereby “moving the mountain rather than the man”). These results emphasise the importance of individual inflammatory history for prognosis and management of ASM hyperplasia in individuals where slow resolution of inflammation is suspected.

A recent study of a horse model of asthma [18] supports these results by comparing the effect of inflammation resolution speed (modulated by corticosteroids) and the role of frequency and severity of environmental stimuli (by avoidance of antigen exposure). Leclere and colleagues have shown that treatment with corticosteroids gives more rapid (compared to pure antigen avoidance) effects on lung function and ASM remodelling [18], which agrees with our model's prediction of the higher importance of rapid inflammation resolution over stimulus magnitude (see Fig. 6a). This prediction is also supported by the acceleration of ASM mass decline by corticosteroids observed in human peripheral airways after a 6-week treatment [25]. Indeed, the ASM cell population can decrease (very slowly) only when μ is below the mild inflammation threshold (Fig. 2b). The larger the inflammation resolution speed, the sooner this long-term recovery regime can be reached.

### Long-term ASM mass accumulation at moderate-to-fast resolution rate

Faster resolution of inflammation decouples individual pro-inflammatory events and thus diminishes the effect of inflammation history in ASM growth. Although this seems to provide greater control over ASM population growth, a mild-to-moderate accumulation of ASM mass over the long term can still occur, provided the magnitude of the exacerbation is above the second threshold μ_2_. The model predicts that long-term ASM mass accumulation is proportional to exacerbation frequency but depends less strongly on exacerbation magnitude (see the growth isolines in Fig. 4). The frequency of the pro-inflammatory events could therefore be considered a secondary target, after ensuring rapid resolution of inflammation. The importance of antigen exposure frequency in the degree of airway remodelling has been also confirmed in animal models [26].

Given that resolution of exacerbations can be affected by current therapies [27] and that clinical factors associated with frequent exacerbations are well recognised [28], mechanism-derived links between these factors and exacerbations could be targeted in future studies to prevent airway remodelling.

### Model limitations and further development

While accounting for the key features of ASM growth, the proposed model neglects many potentially important factors, such as the detailed pathways governing ASM hyperplasia. However, the kinetic parameters for these pathways remain largely unknown. The generality of our model is also a strength, in that its predictions do not depend on the precise nature of the signalling events (leading to the increase in the “inflammatory status”) but only on their dynamical features (magnitude, frequency, resolution speed). In particular, the model is compatible with bronchoconstriction acting as a trigger of airway remodelling [15], [16].

We have not included mechanical interaction of the cells between each other and with the extracellular matrix that could affect the growth and apoptosis rates as well as the total capacity *V* of an airway wall. Our model additionally neglects the spatially heterogeneous and anisotropic growth observed in micrographs and cell hypertrophy. Therefore, the predictions of this ASM growth model are qualitative rather than quantitative.

Extra care should also be taken when interpreting the results of the model in terms of its impact on respiratory lung function. Relative increase in the lumen-narrowing contractile force of an airway wall due to a larger ASM mass could be balanced by a simultaneous increase in the wall stiffness, which may have a constriction-limiting effect [29]. Future models should therefore focus on coupling the mechanics of an airway wall and the ASM population dynamics [29]–[31].

Further experiments to test the model prediction of the role of inflammation resolution rate in co-cultures of inflammatory and ASM cells or in animal models (such as the studies of Garn and co-workers [17], [32]) would be desirable. It would also be of interest to use the model results (in particular, the inflammation severity index) for a detailed statistical analysis of inflammatory status from a time series of inflammatory biomarkers (such as eosinophil counts in sputum or exhaled nitric oxide concentration) and their relation to ASM mass in bronchial biopsies. To our knowledge such longitudinal studies have not yet been performed, due to the difficulties in carrying out repetitive airway biopsies in humans.

## Conclusions

We have developed and analysed a theoretical model of ASM mass accumulation driven by a series of exacerbation events. Our model highlights the importance of the resolution speed of inflammation in long-term management of asthma. It exposes the possibility of false security of strict antigen avoidance or reduced sensitivity to inflammatory stimuli. A few accidental exposures or acute exacerbations at impaired resolution can lead to the accumulation of inflammatory status and hence relatively rapid ASM remodelling.

A more robust anti-remodelling strategy could lie in ensuring the rapid clearance of growth or recruitment factors during the post-exacerbation period, thereby protecting against dangerous build-up of these factors, latent ASM mass increase and reducing the risk of severe remodelling.

## Supporting Information

Material S1 **Supplementary information on the design and solution techniques for the mathematical model.**
(PDF)
