# Supplementary material for: The Role of Inflammation Resolution Speed in Airway Smooth Muscle Mass Accumulation in Asthma: Insight from a Theoretical Model
Source: PLoS One. 2014 Mar 14;9(3):e90162. doi: 10.1371/journal.pone.0090162 (PMC3954558; doi:10.1371/journal.pone.0090162)
Supplement: Material S1 — Supplementary information on the design and solution techniques for the mathematical model. (PDF) [file pone.0090162.s001.pdf]

Supplementary Material

The role of inflammation resolution speed in airway smooth muscle mass accumulation in asthma: insight from a theoretical model

I.L. Chernyavsky[1,*], H. Croisier[1], L.A.C. Chapman[2], L.S. Kimpton[2], J.E. Hiorns[1], B.S. Brook[1], O.E. Jensen[3], C.K. Billington[4], I.P. Hall[4], S.R. Johnson[4]

S1 Supplementary Methods

This section gives additional technical details about the design and solution techniques for the model.

S1.1 Model Assumptions and Formulation

Consider the following system of ordinary differential equations, describing the dynamics of two subpopulations of ASM cells defined in Fig. 2(a,b):

$$\dot{p} = \lambda_p \, p \left(1 - \frac{p+c}{V}\right) - \lambda_{pc} \, p + \lambda_{cp} \, c \,, \tag{S1a}$$

$$\dot{c} = \lambda_{pc} \, p - (\lambda_{cp} + \lambda_a) \, c \,, \tag{S1b}$$

where p and c are the size of p- and c-subpopulations respectively (e.g. measured in number of cells per cross-sectional airway wall area), a dot over a variable represents its rate of change (a time derivative), λ_p is the proliferation rate, λ_a is the apoptosis rate, and λ_{pc}, λ_{cp} are the switching rates, and V is the total capacity (maximal population size, measured in the same units as p and c). The system (S1) is subject to the initial conditions $p|_{t=0} = p_0$ and $c|_{t=0} = c_0$. The total size of the ASM population is thus given by

$$s = p + c \,. \tag{S2}$$

We incorporate the effect of short-term inflammatory events by assuming that the switching rate λ_{cp} can change according to the inflammatory status μ which, for instance, could represent airway eosinophil cell count in sputum per unit volume. The dynamics of μ is described by the following equation:

$$\dot{\mu} = -\lambda_d \, \mu + a \, f(t;\omega) \,, \quad f(t;\omega) = \sum_i \delta(t - t_i), \tag{S3}$$

[*] Author for correspondence (Igor.Chernyavsky@nottingham.ac.uk).
[1] School of Mathematical Sciences, University of Nottingham
[2] Mathematical Institute, University of Oxford
[3] School of Mathematics, University of Manchester
[4] Department of Therapeutics and Molecular Medicine, University of Nottingham

where δ denotes Dirac's δ-function, t_i is the time of an acute inflammatory event, λ_d is the decay rate of the inflammatory factor, a is the magnitude of a single acute inflammatory event, and $\omega = 1/\mathbb{E}[t_{i+1} - t_i]$ is the mean event frequency.

All parameter ratios are expressed in terms of a small parameter $\varepsilon \equiv 1/(\lambda_p T)$, which is defined as the ratio of the proliferation timescale $1/\lambda_p$ to the remodelling timescale T; Table 1 gives the scalings of the corresponding ratios of rates used in the model. It should also be noted that the choice of the relative orders of magnitude for the rates (Fig. 2b) is not unique; however, a global rescaling of all the parameters by a power of ε does not affect our main conclusions. Also, similar results could be obtained by assuming that λ_{pc}, rather than λ_{cp}, or their ratio, are functions of μ.

S1.2 Solution Techniques

We use a combination of linear stability analysis, two-time-scale asymptotics and numerical simulation to characterise qualitatively the dynamics of a population of ASM cells. Direct numerical simulation of (S1)–(S3) is performed with `Matlab` `ode45` Runge-Kutta solver at the relative tolerance of 10^{-6}, where individual inflammatory events in (S3) are approximated as a series of Gaussian "peaks" $f(t) \approx \sum_i \exp\{-\lambda_p^2\,(t-t_i)^2/(2\sigma^2)\}\,/\sqrt{2\pi\sigma^2}$ for $\sigma = 0.01 \ll \lambda_p/\omega$.

ASM growth dynamics

For the given assumptions, equations (S1) lead to three distinct asymptotic growth regimes for a population of ASM cells.

The first regime (case (1) in Fig. 2(b,d)) is characterised by an approximately constant c-subpopulation and negligible p-subpopulation when the balance between the state-switching, proliferation and apoptosis

$$\frac{\lambda_{cp}}{\lambda_{pc}} \sim \frac{\lambda_a}{\lambda_p} \tag{S4}$$

is satisfied; in the second growth regime (case (2) in Fig. 2(b,d)), the c-subpopulation of ASM cells exhibits a slow logistic growth, which is significant only in the long term (i.e. months to years), while the p-subpopulation remains small; in the third regime (case (3) in Fig. 2(b,d)), the ASM cell population splits into the c- and p-subpopulations of comparable size that grow logistically in relatively short time-scales (i.e. weeks to months).

Considering the dynamics of the inflammatory status μ for the moderate-to-large speed of inflammation resolution ($\mathrm{IR} \equiv \lambda_d/\lambda_p \gtrsim 1$), an approximate solution to (S3) takes the form of a series of independent 'spikes' (Fig. S1):

$$\mu(t) \approx a \sum_{i=0}^{n} \mathrm{e}^{-\mathrm{IR}\,\lambda_p\,(t\,-\,t_i)}\,, \tag{S5}$$

where $n \equiv \lfloor \omega\, t \rfloor$ ($\lfloor x \rfloor$ denotes the integer part of x).

Since the time spent by an ASM cell population above the "severe" inflammation sensitivity threshold μ_2 has the greatest impact on the net population growth, we estimate this time $T_{\text{severe}} = N\Delta t$ as the product of the number of events $N = T\omega$ over a fixed long-term observation period T and the time interval Δt spent above μ_2 after each exacerbation of given magnitude

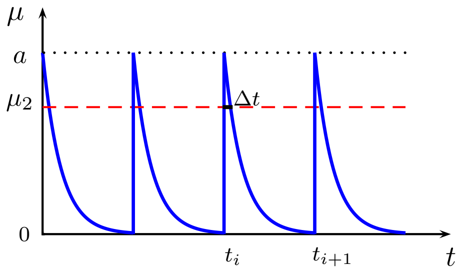

Figure S1. Schematic of the inflammatory status dynamics μ (solid) given by (S5) for large IR and $a > \mu_2$. Dashed red line indicates the "severe" inflammatory threshold μ_2; thick black solid denotes the time-interval Δt spent above the threshold.

$a > \mu_2$. The growth isoline of total ASM cell population $s(T) = \text{const}$ (see Figs 4 and 6a) can thus be approximately characterised by $T_{\text{severe}}/T \equiv \omega\,\Delta t = \text{const}$. In particular, for a single exacerbation, from (S5) we have $\mu_2 \approx a\,\mathrm{e}^{-\mathrm{IR}\,\lambda_p\,\Delta t}$, and the time interval is $\Delta t \approx (\lambda_p\,\mathrm{IR})^{-1}\,\log\frac{a}{\mu_2}$, subject to the compatibility condition $(0 < \Delta t \ll \omega^{-1})$. The isoline of the ASM population growth for fast inflammation resolution is thus given by

$$\mathrm{IR} \approx A\left(\frac{\omega}{\lambda_p}\right)\log\left(\frac{a}{\mu_2}\right), \quad \mathrm{IR} \gg 1\,, \tag{S6}$$

where a/μ_2 and ω/λ_p are the relative magnitude and mean frequency of exacerbations respectively, and $A \equiv T/T_{\text{severe}} \geq 1$ is a constant that defines the value of the fold-increase in ASM population size. The predicted growth isoline (S6) is plotted as the dashed white lines in Figure 4.
